# Implementation and performance of haemovigilance systems in 10 sub-saharan African countries is sub-optimal

**DOI:** 10.1186/s12913-021-07235-0

**Published:** 2021-11-20

**Authors:** Washington T. Samukange, Verena Kluempers, Manvi Porwal, Linda Mudyiwenyama, Khamusi Mutoti, Noel Aineplan, Helga Gardarsdottir, Aukje K. Mantel-Teeuwisse, C. Micha Nuebling

**Affiliations:** 1 Division of Pharmacoepidemiology & Clinical Pharmacology, Faculty of Science, Utrecht Institute for Pharmaceutical Sciences (UIPS), Utrecht University, Utrecht, the Netherlands; 2 Paul Ehrlich Institut, Langen, Germany; 3 Medicines Control Authority of Zimbabwe (MCAZ), Harare, Zimbabwe; 4 South African Health Products Regulatory Authority (SAHPRA), Pretoria, South Africa; 5 National Drug Authority (NDA), Kampala, Uganda; 6 Major Policy and International Relations, Paul Ehrlich Institut, Langen, Germany

**Keywords:** Haemovigilance, Blood Safety, Global Benchmarking Tool, Blood Regulatory Systems

## Abstract

**Background:**

Haemovigilance is an important element of blood regulation. It includes collecting and evaluating the information on adverse events resulting from the use of blood and blood components with the aim to improve donor and patient safety. We describe the results of the pilot of the integrated GBT+ Blood for the haemovigilance function in 10 sub-Saharan African countries.

**Methods:**

We piloted the integrated WHO Global Benchmarking Tool plus Blood (GBT+ Blood) to assess the haemovigilance function of national regulatory authorities (NRAs) in Ethiopia, Kenya, Malawi, Nigeria, Liberia, Rwanda, South Africa, Tanzania, Uganda, and Zimbabwe. Data obtained from documents and face to face interviews were used to determine the status of implementation and performance of the following six indicators; legal provisions regulations and guidelines, organisation and governance, human resources, regulatory processes, transparency and accountability and finally, monitoring progress and assessing impact, by estimating median scores across 20 sub-indicators. In addition, a cluster analysis was performed.

**Results:**

The countries showed inter-organisation variability in implementation and performance of the haemovigilance function. The overall median score (all sub-indicators) was 44 % (range: 7.5 % - 70 %). The lowest average performance scores were for the arrangement for effective organisation and coordination (35 %) and human resources (35 %) indicators. The highest average scores were observed for the mechanism to promote transparency and mechanism to monitor regulatory performance indicators (50 % and 60 %, respectively). We identified clusters of best-implemented sub-indicators from the procedures for haemovigilance and poorly implemented sub-indicators from the legal provisions, regulations and guidelines for haemovigilance and human resources.

**Conclusions:**

Implementation of sub-indicators and performance of haemovigilance systems varied greatly for all countries with a few countries performing reasonably well in the implementation of some sub-indicators under procedures for haemovigilance. Most countries were poorly implementing sub-indicators in the legal provisions, arrangement for effective organisation and human resources indicators. The legislative provisions in most countries were at a nascent stage. There is a need to set up targeted and customised technical support coupled with prioritised interventions to strengthen the capacities of NRAs.

**Supplementary Information:**

The online version contains supplementary material available at 10.1186/s12913-021-07235-0.

## Introduction

It is widely acknowledged that access to quality-assured blood and blood components improves health and saves lives in acute emergencies [1, 2]. Blood transfusion when used appropriately is very safe and effective [2]. A major public concern with regards blood and blood products in African countries are the risks of safety as a result of the high rates of transfusion transmissible infectious diseases in the general population. The tragic scandals of transfusion-transmitted AIDS infections and transfusion transmissible newly emerging blood-borne diseases like Zika have led to increased blood safety concerns [2]. There are several factors calling on governments to establish mechanisms for the systematic surveillance of complications of blood transfusions through nationally organised and coordinated haemovigilance systems, which were not established at the time of the blood scandals [2].

Haemovigilance is defined as ‘a set of surveillance procedures covering the whole transfusion chain (from the collection of blood and its components to follow up of recipients)’ [1]. It is intended to collect and assess information on unexpected or undesirable effects resulting from the therapeutic use of labile blood products, and to prevent their occurrence or recurrence [2]. The ultimate goal of haemovigilance is the continuous improvement of the transfusion chain through corrective and preventive actions to improve patient safety outcomes, enhance donor safety and reduce wastage [3]. This is achieved through informed transfusion policies, improved standards and guidelines, and early warning of new complications [4, 5].

Haemovigilance is a relatively recent development in transfusion safety and has since its introduction become an integral part of blood safety worldwide [5]. The concept of haemovigilance first came into existence in France in 1991. Since then, several countries have established haemovigilance systems (e.g. Germany 1994, Greece 1995, and UK 1996) [3]. There are significant differences in haemovigilance systems globally, in terms of definitions, terminology, standardised reporting, organisational schemes, state of development and implementation [1, 3], mandates and responsibilities [6]. The latter two are often unclear and undefined, respectively [1, 3]. Other challenges experienced by countries in setting up a functioning haemovigilance system include funding shortages, low sensitivity [3], insufficient training of personnel [8], and hesitation to move forward by implementing authorities.

To ensure public confidence in the safety of blood products, the strengthening of blood safety monitoring is, therefore, a key need. This need was recognised and identified at a global level and was pronounced by two World Health Assembly (WHA) resolutions, namely WHA63.12 (2010) on ‘Availability, safety and quality of blood products’ and WHA 67.20 (2014) on ‘Strengthening Regulatory Systems’ in WHO member states [2, 9]. In response to the latter resolution WHO developed the Global Benchmarking Tool (GBT) for medicines and vaccines that would evaluate national regulatory systems, generate and analyse evidence of regulatory system capacity and performance, facilitate the formulation and implementation of institutional development plans and provision of technical support to national regulatory authorities (NRAs) and governments [6, 10]. Furthermore, the WHO Blood Regulators Network (BRN) developed criteria for independent and uniform assessment (or self-assessment) of blood regulation in different parts of the world [11].

The subsequent process of integrating the WHO Criteria for Assessment of Blood Regulatory Systems (for blood, blood components, plasma-derived medicines, and medical devices including in-vitro diagnostics) into the GBT began in 2017 and resulted in the comprehensive WHO GBT+ Blood covering all relevant blood regulatory functions. The GBT+ Blood provides a basis to identify regulatory capacity and performance and barriers for NRAs to meet minimum regulatory functioning goals. It also reflects current global best practices in blood regulation and allows the collection of quantitative and qualitative data which can be analysed simultaneously to give a context-specific interpretation of regulatory capacity and performance over time.

The aim of this manuscript is to describe the results of the pilot of the integrated GBT+ Blood for the haemovigilance function in 10 sub-Saharan African countries. We identified and compared the status of implementation and performance of haemovigilance systems in those 10 countries. We further identified haemovigilance system strengths, gaps and challenges and defined actions that can be undertaken to strengthen safety monitoring of blood and blood products in the coming years in Africa.

## Methods

### Study design

This was a cross-sectional descriptive study to look at the existing haemovigilance systems in 10 Sub-Saharan Africa countries, namely Ethiopia, Nigeria, Malawi, Kenya, Liberia, Rwanda, Uganda, South Africa, Tanzania, and Zimbabwe. The NRAs that were selected were English-speaking, and were located in three geographical clusters; West Africa (*n* = 2), East Africa (*n* = 5), and Southern Africa (*n* = 3). The benchmarking process followed the previously described 13-step method by van Lent et al. (see Supplementary Table ST1) [12]. The first five of these thirteen steps involved the identification of the problem, forming the benchmarking team, choosing the benchmark partners, and defining their main characteristics, and identifying the relevant stakeholders. We describe here in the methodology section the next steps (6 to 12). Step 13, implementation of the improvement plans, is outside the scope of the present study.

### The Integrated WHO Global Benchmarking Tool+ Blood

The WHO Global Benchmarking Tool was designed by WHO as a standardised benchmarking and assessment tool to evaluate and measure regulatory performance, initially for vaccines and medicines and now expanded to include blood and blood products [6]. The GBT+ Blood is used to evaluate and measure the performance of each regulatory function, that is national regulatory system, marketing authorisation and registration, haemovigilance, licensing of blood establishments, market control and post-marketing surveillance, regulatory inspections, clinical trial authorisation, lot release and lab access, approval of blood (product/process) and approval of medical devices and associated substances and in-vitro diagnostic (IVDs) medical devices. The benchmarking tool is revised regularly through informal and formal consultations of national and regional regulatory experts as well as public consultations to keep the tool fit for purpose.

### Framework and indicators

The WHO BRN Assessment Criteria for Blood Regulatory Systems [10] and the GBT (medicines and vaccines) provided the framework and structure for indicators and sub-indicators for the haemovigilance function in the GBT+ Blood as described under step 6. To ensure structural consistency among the different regulatory functions in the GBT+ Blood, the sub-indicators are grouped according to the following six specific themes or indicators, (1) legal provisions, regulations and guidelines, (2) organisation and governance, (3) human resources, (4) regulatory processes, (5) transparency and accountability and finally (6) monitoring progress and assessing impact [6]. The set of indicators and sub-indicators integrated into the GBT+ Blood was agreed upon by a sub-group of experts from the WHO BRN (steps 7 and 8) in August 2017 in Geneva, Switzerland. The integrated haemovigilance function had a total of 25 sub-indicators spread across the six indicators and are shown in Supplementary Figure SF1 and Supplementary Table ST2.

The WHO GBT also incorporates the maturity level concept from the International Standard Organisation (ISO) 9004:2018 [13] (Supplementary Figure SF2). This concept enables the assessment of the status and performance of regulation with a variety of indicators and sub-indicators and gives an overall view of the NRA’s maturity based on the achievement of general benchmarks in regulatory practice. The maturity levels for the sub-indicators are distributed as shown in Supplementary Table ST2; 4 sub-indicators for maturity level 1, 2 sub-.indicators for maturity level 2, 14 sub-indicators for maturity level 3, and 5 sub-indicators for maturity level 4. For purposes of this study, we used only the 20 sub-indicators for maturity levels 1-3, and left out 5 sub-indicators excluding maturity level 4 sub-indicators as data for these sub-indicators were not consistently collected in all countries. Complying with all included 20 sub-indicators would result in the benchmarked haemovigilance system receiving a rating of a stable, well-functioning and integrated system at maturity level 3.

### Benchmarking methodology and data collection

Before visiting the participating NRAs, authorisation and approval for the benchmarking was sought from the heads of agencies by the African Union Development Agency – African Medicine Harmonisation Programme (AUDA – AMRH) via e-mail. Key individuals with overall responsibility and knowledge of the respective national system in each country were identified. They were informed about the assessment and asked to share the legal and statutory documents and other relevant information with the external assessment team before the benchmarking visit. The documents requested were extracts of national legislation describing responsibilities of the relevance of the function of the national haemovigilance system and pharmacovigilance or haemovigilance regulations and guidelines.

A priori data from the previous self-benchmarking by the NRA was also sent to the benchmarking team, where it was available and was used to pre-fill the sub-indicators before each visit. The actual benchmarking assessment was carried out on-site at each of the 10 NRA’s premises with the NRA’s team. The face to face assessment interviews were done with at least two senior staff from the vigilance and safety teams of the national regulatory agencies. The assessment and data collection in the 10 countries were conducted from February to August 2018 (Step 9).

The benchmarking principles on assessment procedures and conducting benchmarking assessments (how to score, evidence to review) that are enshrined in the WHO Manual for benchmarking of the national regulatory system of medical products were applied [6, 7]. Using the integrated WHO GBT+ Blood haemovigilance indicators as a guide, questions and follow-up questions were asked as required until the assessors had gained enough understanding of the availability and functionality of the relevant structure, process, systems, and outcomes. The responses were recorded in the data collection module of the WHO GBT+ blood. To increase the validity of the data, the outcomes were presented to the key individuals of the NRA for verification and confirmation.

### Data analysis

The data from each country assessment were collected in the Microsoft Access (Microsoft Corporation, Redmond, WA, USA) based WHO GBT+ Blood data collection module and exported into a Microsoft Excel (Microsoft Corporation, Redmond, WA, USA) template from the WHO GBT+ with all six haemovigilance indicators (Step 10).

To determine whether a sub-indicator was implemented or not, the NRA had to provide documentary evidence and references when implementing the sub-indicator. When documentary evidence such as legislation (Act or Regulation), policy, and/or guidelines that were being implemented and enforced were available, the sub-indicator would be scored ‘Yes’ and the system would give a numerical score of 1 [6, 7]. When the NRA had documentary evidence (such as legislative provisions, policy, guidelines or procedures) without any further evidence of implementation or still at the initial stages of implementation of their legal requirements, the sub-indicator was scored ‘Partial’ and the system scored the sub-indicator with a score of 0.5. When the NRA was not implementing the sub-indicator or had neither documentary evidence nor references to satisfy the requirement of the sub-indicator, then the sub-indicator was scored ‘No’ and the system would give this a numerical score of 0.

To determine the status of implementation of haemovigilance sub-indicators in each country, the sum of the sub-indicator scores were expressed as a percentage of the maximum score that could be obtained. Similarly, to determine the performance of specific haemovigilance functions, the sum of sub-indicator responses for each indicator were analysed. The maturity levels of the haemovigilance systems of each country were analysed by comparing the sum of the responses to each of the sub-indicators against their maturity levels. To segment and identify homogenous groups of sub-indicators (or indicators) and their performance from the GBT+ Blood haemovigilance function, hierarchical cluster analysis was performed using SPSS version 26. The sub-indicator responses were categorized as ‘Yes’ or ‘No’, with ‘Partial’ counted as ‘Yes’ for purposes of this analysis. A dendrogram was generated to categorise clusters of homogenous groups of sub-indicators (and their responses). The authorities were anonymized randomly for the presentation of results.

## Results

### General characteristics of the benchmarked national regulatory agencies

The characteristics and profiles of the benchmarked national systems are shown in Table 1. There were structured national blood transfusion services (*n* = 9) and a national blood programme (*n* = 1) with the national mandate to collect and supply blood. Blood donations were achieved through 100 % voluntary non-remunerated blood (*n* = 6) and family replacement blood (*n* =4) collections. The national blood transfusion services were collecting over 2 000 000 units of blood annually, with South Africa responsible for almost 50 % of those collections.

**Table 1:** Characteristics and profiles of benchmarked national systems

Country	NRA	Blood System	Blood covered by legislation	Annual Blood Collections (units)	Collections from	Blood Components Prepared
Ethiopia	Ethiopian Food and Drug Administration (EFDA)^1^	National Blood Bank Service and Red Cross Society	✓	173 923	VNRD FRD	Whole blood
Kenya	Pharmacy and Poisons Board (PPB)	Kenya National Blood Transfusion Service	✓	167 100	VNRD	Whole blood, Red Cell Concentrates, Platelets, Fresh Frozen Plasma
Liberia	Liberia Medicines and Healthcare Products Regulatory Authority (LMHRA)	Blood Safety Program, Ministry of Health	✓	35 000	VNRD FRD	Whole blood
Malawi	Pharmacy Medicines and Poisons Board of Malawi (PMPB)	Malawi National Blood Transfusion Service	✓	80 000	VNRD	Whole blood, Red Cell Concentrates, Platelets, Fresh Frozen Plasma, Cryoprecipitate
Nigeria	National Agency for Food and Drug Administration and Control	National Blood Transfusion Service of Nigeria, Regional and State Blood Transfusion Services	✓	50 000	VNRD FRD	Whole blood
Rwanda	Rwanda Food and Drugs Authority (RFDA)^2^	National Centre for Blood Transfusion	✓	68 695	VNRD	Whole blood, Red Cell Concentrates, Platelets, Fresh Frozen Plasma
South Africa	South African Health Products Authority (SAHPRA)	South African National Blood Service and Western Cape Blood Service	✓	929 000	VNRD	Whole blood, Red Cell Concentrates, Platelets, Fresh Frozen Plasma
Tanzania	Tanzania Medicines and Medical Devices Authority (TMDA)^3^	National Blood Transfusion Service Tanzania and Regional Hospitals	✓	196 735	VNRD FRD	Whole blood, Red Cell Concentrates, Platelets, Fresh Frozen Plasma
Uganda	National Drug Authority (NDA)	Uganda Blood Transfusion Services	✓	239 000	VNRD	Whole blood, Red Cell Concentrates, Platelets, Fresh Frozen Plasma
Zimbabwe	Medicines Control Authority of Zimbabwe (MCAZ)	National Blood Services Zimbabwe	✓	65 126	VNRD	Whole blood, Red Cell Concentrates, Platelets, Fresh Frozen Plasma, Cryoprecipitate

^1^formerly Ethiopian Food, Medicine and Healthcare Administration, ^2^formerly Department of Pharmaceutical Services, Rwanda and ^3^formerly Tanzania Food and Drug Authority

VNRD – voluntary non-remunerated donation

FRD – family replacement donation

The NRAs each had independent or partially independent NRAs (*n* = 9) or had a department in the Ministry of Health (*n* = 1), all with the legal mandates to regulate medicines including blood, blood components and blood products (Table 1). Some of the authorities were yet to establish or were at the initial stages of setting up vigilance and/or medicine safety monitoring teams in their agencies at the time of the benchmarking (*n* = 3).

### Overall performance of national haemovigilance systems

**Fig. 1:**
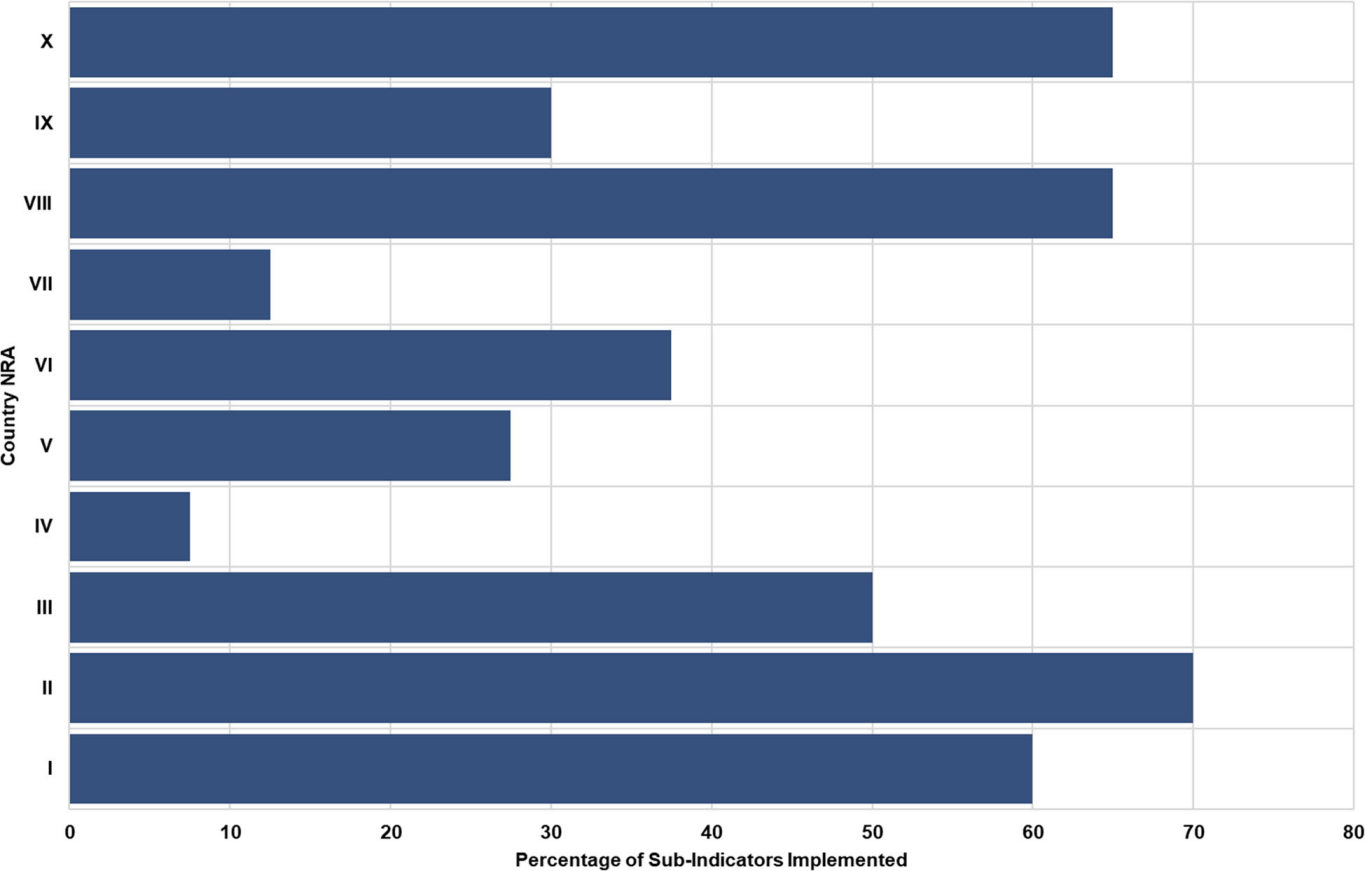
Overall Implementation of Haemovigilance function in benchmarked country NRAs

**Fig. 2:**
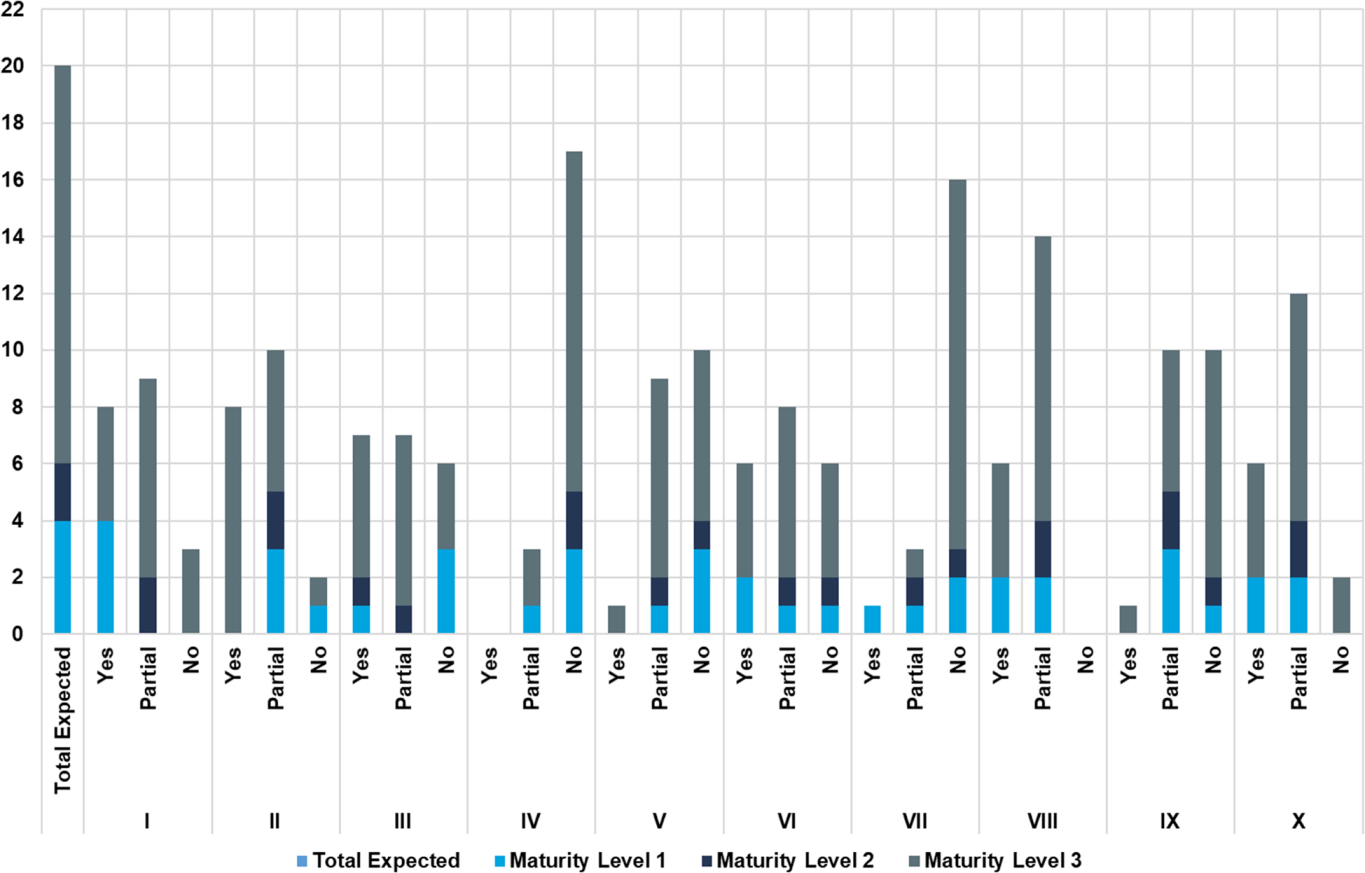
Cumulative performance of 20 sub-indicators for each maturity level in all the benchmarked country NRAs

**Fig. 3:**
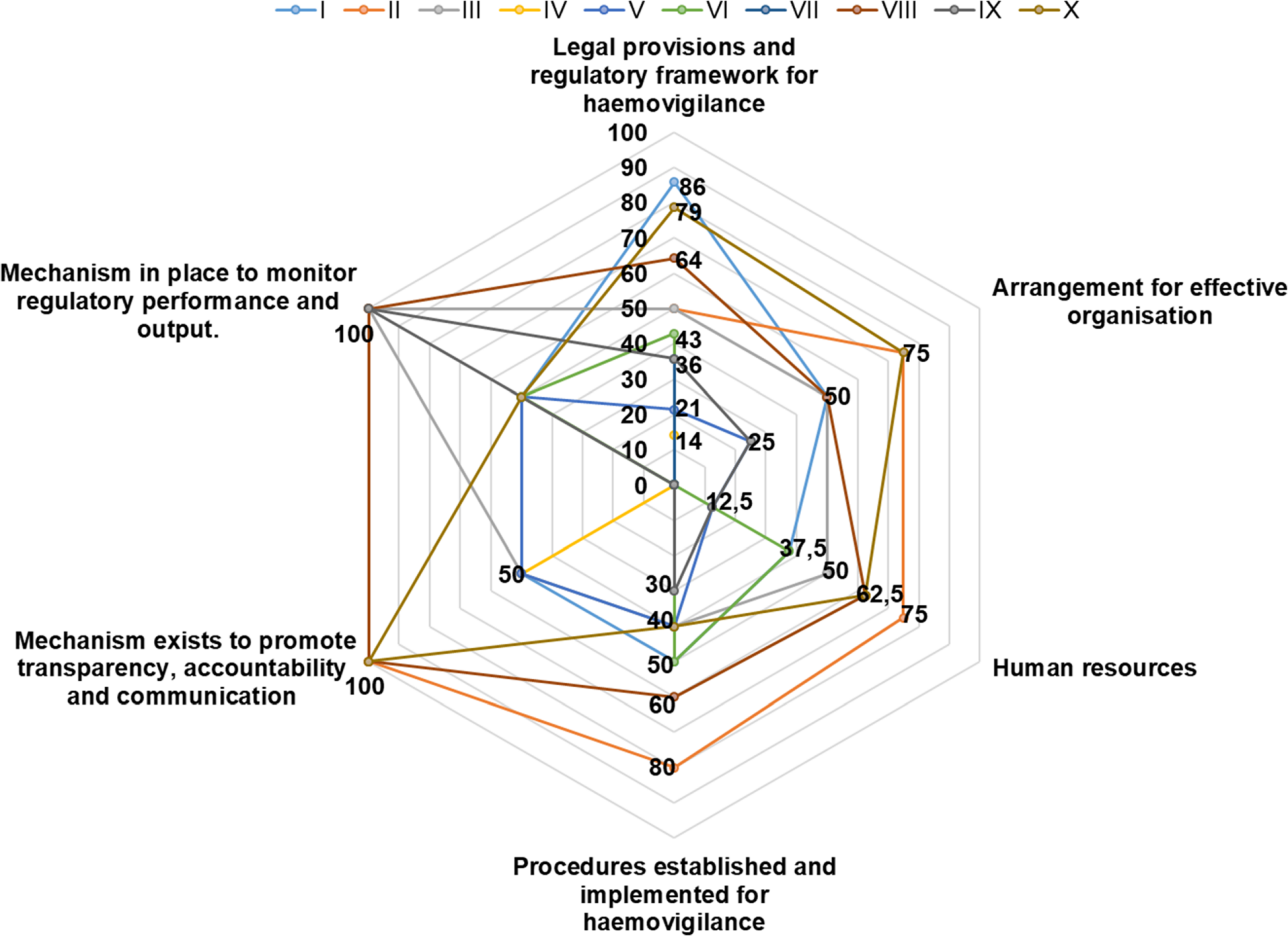
Performance of haemovigilance indicators in 10 NRAs

**Fig. 4:**
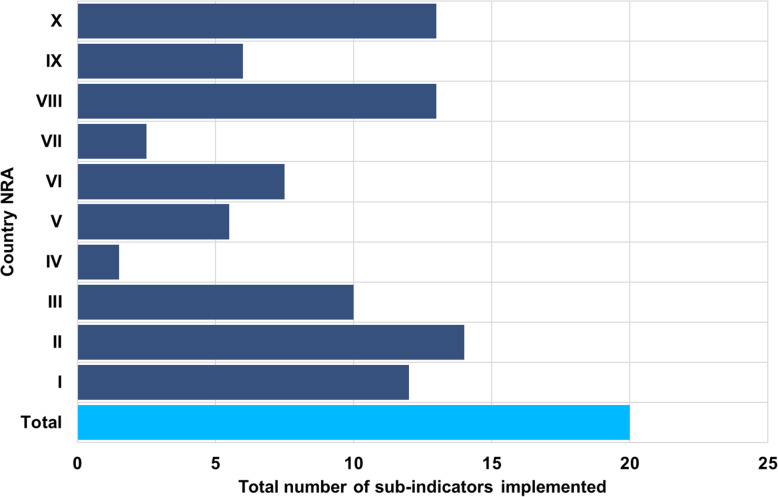
Performance of NRAs on implementation of all sub-indicators

**Fig. 5:**
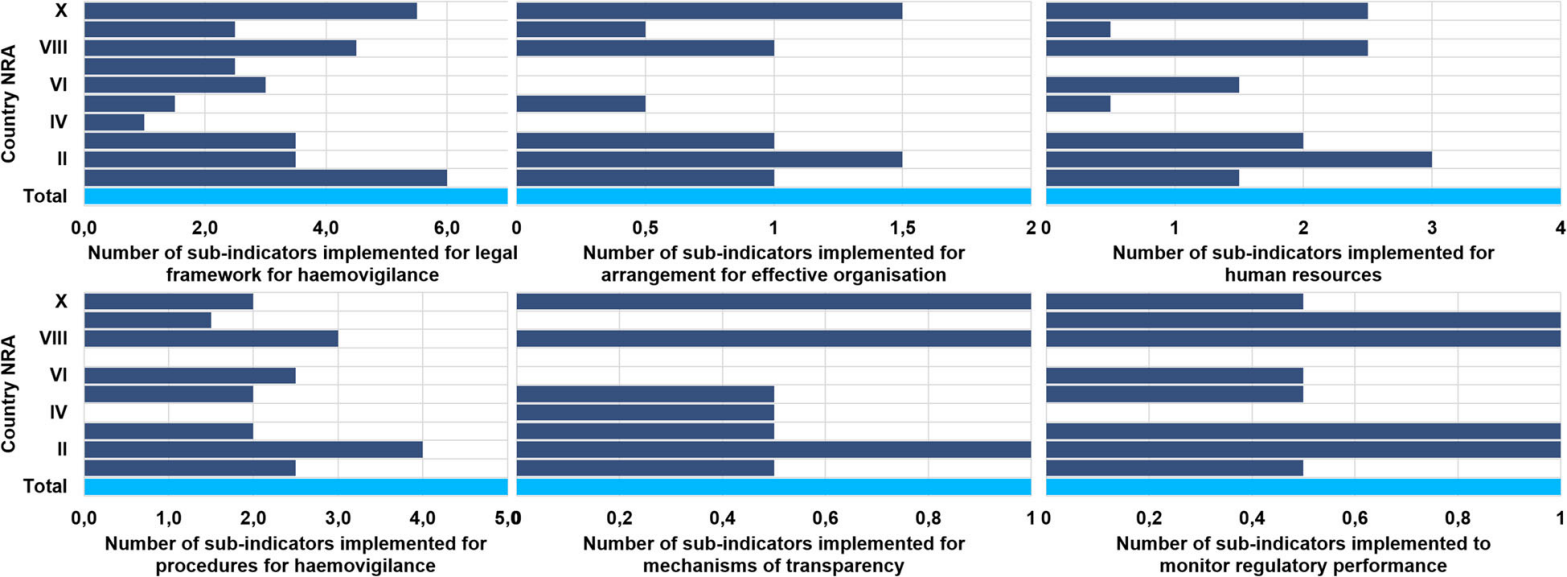
Performance of NRAs on each indicator and sub-indicator

The median overall score for implementation of the haemovigilance function in all countries was 44 % (range: 7.5 – 70 %). The highest score for implementation of the haemovigilance function was 70 % and only obtained in one country (Fig. 1). None of the benchmarked NRAs were implementing and performing haemovigilance at maturity level III (Fig. 2). Only one country was implementing all four maturity level I sub-indicators. A few countries were implementing some maturity level III sub-indicators with a few or none from the lower maturity levels being implemented at all. The performance of national haemovigilance systems in the participating NRAs varied greatly across the six indicators (Fig. 3). Figures 4 and 5 provide an overview of the performance across all sub-indicators and more detailed information per indicator is given below.

### Indicator 1: Legal provisions, regulations and guidelines required to define the regulatory framework for haemovigilance

The average performance score for this indicator for all participating NRAs was 48 % (range 14.3 % - 87.7 %). Although all authorities had medicine legislation that mandated each NRA to fully develop, implement and coordinate their medicine safety monitoring and surveillance systems, most authorities (*n* = 9) lacked specific regulations, and/or national haemovigilance policies mandating (or voluntarily) the reporting of adverse reactions and adverse events from the processing and transfusion of blood and blood components. Only one national system had regulations requiring the reporting of adverse reactions for blood transfusion, but these were not comprehensive enough. None of the countries had comprehensive national guidelines for haemovigilance.

### Indicator 2: Arrangement for effective organisation and good governance

Two systems scored high since they had mechanisms and strategies to coordinate stakeholders at the national level (the blood transfusion services and the national regulatory authorities). Most benchmarked systems (*n* = 8) were lacking effective mechanisms to coordinate stakeholders. The average performance score for this indicator was 35 % (range: 0 – 75 %). Interactions among stakeholders involved in blood safety was limited and fragmented, with three countries having some kind of platform or strategy for interaction between the national blood service and the NRA. The legislative mandate and responsibility for setting up and coordinating national haemovigilance systems in all the 10 countries belonged to the NRAs. However, five national blood transfusion services had either set up a system for the collection of spontaneous reports of adverse reactions for blood transfusions or were at the initial phases of setting up. These activities were not known to the NRAs and other stakeholders and were not linked to the overall national system for medicine safety monitoring in those countries.

### Indicator 3: Human Resources

All the benchmarked NRAs lacked adequately trained staff in blood safety and haemovigilance, and therefore had limited capacities to perform haemovigilance activities. The highest complement of full-time and part-time staff observed in the medicine safety monitoring team at any NRA was 10 persons. All countries reported that they did not have adequate numbers of staff in their medicine safety monitoring teams, with three NRAs not having any staff assigned to this team. This was also evident from the fact that none of the authorities had assigned haemovigilance responsibilities to their teams. Similarly, while a number of the benchmarked authorities had national medicine safety advisory committees in place, haemovigilance activities were not considered part of their activities at the time of the benchmarking. Overall, a mean performance score of 35 % (range: 0 – 75 %) was observed for this indicator.

### Indicator 4: Procedures for haemovigilance

The analysis of the established system for the regular review of safety and effectiveness activities as part of the vigilance system revealed the extent to which standard processes and procedures were being implemented in each country. The mean performance score for this indicator was 39 % (range: 0 – 80 %). Most NRAs (*n* = 7) had well-developed spontaneous reporting systems with established forms for collection, assessment, investigation, interpretation and response to reports of adverse reactions from medicines and vaccines. The system for collection and assessment of reports of adverse reactions from blood transfusion was not in place in all NRAs, while only one national blood transfusions service had spontaneous reporting forms and procedures in place for reporting adverse reactions from blood transfusions. Adverse events (errors, incidents) and active surveillance activities were not included in any of the authorities that were benchmarked.

### Indicator 5: Mechanisms to promote transparency, accountability and communication

Each of the authorities was assessed against their mechanisms to ensure communication and transparency within the NRA, and with the public including international partners. Three countries had well-developed mechanisms to communicate internally within their NRAs while being transparent with the public through several mechanisms which included regularly updated websites and other media.

### Indicator 6: Mechanisms to monitor regulatory performance and output

Four countries had well-developed mechanisms to monitor regulatory action and decision making using vigilance information, but there was no coordination between the national blood services and the NRAs. There were no regulatory actions linked to haemovigilance information in those countries where some haemovigilance surveillance was already in place.

### Sub-indicator cluster analysis

**Fig. 6:**
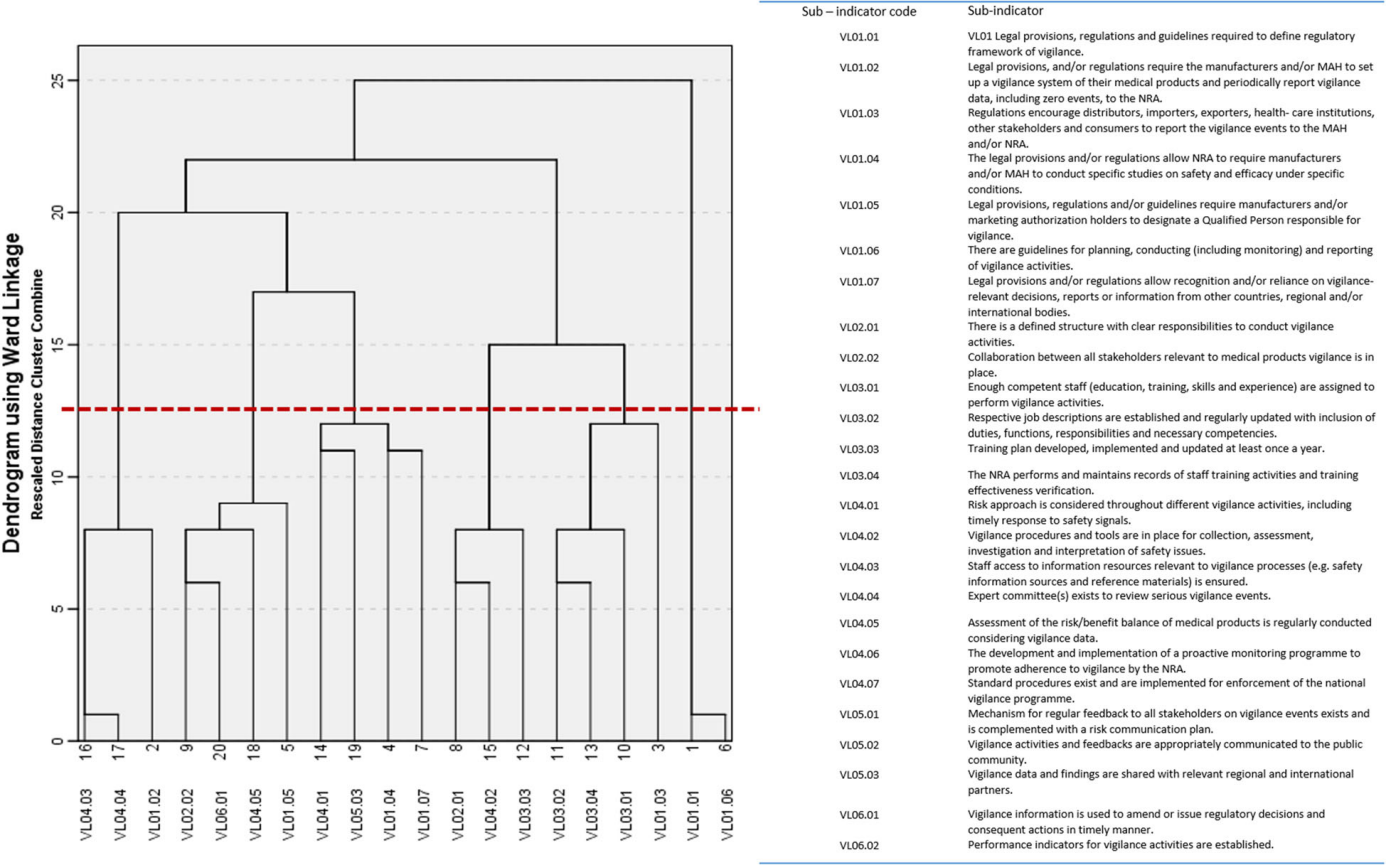
Hierarchical clustering of responses to all sub-indicators (Only clusters below the red line were considered)

Six clusters emerged from the hierarchical cluster analysis (Fig. 6). Cluster I (sub-indicators 16, 17, 2) contained the best-implemented sub-indicators, mostly from the procedures for haemovigilance. Cluster V (11, 13, 10, 3) and VI (1, 6) contained the least implemented sub-indicators, mostly from the human resources and legal provisions indicators. Clusters II, III, and IV are a mix of sub-indicators from different indicators (left to right on the Dendrogram) which shift from good implementation to poor implementation.

## Discussion

We documented the performance of the haemovigilance function in national blood regulatory systems in ten Sub-Saharan African countries. The GBT+ Blood was used in a pilot in 10 sub-Saharan African country NRAs for identifying haemovigilance system strengths, gaps and challenges and defined actions that can be undertaken to strengthen safety monitoring of blood and blood products. Our findings show that there was a good implementation of a few sub-indicators belonging to the procedures for haemovigilance and the legal provisions, regulations and guidelines for haemovigilance indicators and especially poor implementation of all sub-indicators from the human resources indicator. A few countries achieved excellent performances in a few sub-indicators and indicators but had mediocre performances in other sub-indicators. None of the NRAs was implementing all 20 sub-indicators and therefore none of the haemovigilance systems could be described as stable, well-functioning and integrated haemovigilance systems performing at maturity level 3.

Substantial gaps such as lack of a comprehensive legal framework for haemovigilance outside of the basic medicine legislation, lack of clear understanding and distinction of the function of the blood service, the hospital blood banks and the NRA and a lack of human resources were observed to be the main drawbacks in the implementation and performance of haemovigilance systems of all countries. This finding converges with previous studies on haemovigilance systems in developing countries [4, 8]. The existence of basic legislation demonstrates a high-level commitment by all countries to improve the safety of medicines. However, the lack of development and implementation of more specific and comprehensive haemovigilance regulations reflects fundamental limitations in enforcing and monitoring blood safety [14, 15] and suggests a hesitation to move forward by implementing strong actions by policymakers [15]. Legislation to regulate blood transfusion in several countries developed in response to significant public pressure on policymakers following the impact of HIV and hepatitis transmissions through transfusion, which is perhaps a missing factor in the countries included in this study [5, 16]. The variation between national provisions, stakeholder coordination, and haemovigilance implementation processes reflect political and legislative differences, and differences in the blood safety development landscape among countries.

We noted that, despite the significant progress made in several countries, including some parts of Europe and Japan since the early 1990 s [17], haemovigilance has remained a fairly new concept despite the global recognition accorded to it through many WHA resolutions [2]. The benchmarked countries in this study are no exception to this general notion. The growth of haemovigilance in the benchmarked countries lacked focused advocacy and technical support equivalent to that observed with pharmacovigilance through the WHO Programme for International Drug Monitoring (PIDM) whose main aim is to develop a comprehensive global pharmacovigilance strategy to respond to the healthcare needs of low- and middle-income countries in monitoring medicine safety. The recently launched WHO Action Framework to Advance Universal Access to Safe, Effective and Quality-Assured Blood Products is a renewed effort to scale up efforts to support the implementation of effective surveillance and haemovigilance systems in the WHO Member States [9].

The slow evolution of haemovigilance in the benchmarked countries raises uneasy questions over the types of blood and blood component related safety incidents that may have gone and are still going unnoticed [18]. The sub-optimal implementation of haemovigilance compared to pharmacovigilance is postulated to put blood donors, blood quality and safety, and blood transfusion patients at risk. It can be inferred from our study that in the absence of more organised, standardised and systematic surveillance systems several transfusion-related events may not be picked up in most of the countries in this study. It is common to observe clinical manifestations of medicine-related harm in healthcare systems [19]. One of the best ways to influence policymakers and political leaders on the need for haemovigilance in their countries is through the evidence on the burden of blood transfusion-related harm in their populations [20]. Unfortunately, very few studies have documented the burden of blood transfusion-related harm in the assessed countries.

It was observed that the human resources sub-indicators obtained the worst performance scores in the majority of the benchmarked NRAs. The capacity for reporting, collecting and analysing haemovigilance data in most benchmarked countries was insufficient. It has been widely reported that health systems (including NRAs) in developing countries lack expertise, resources (especially funding and capacity), and training programmes to fully implement comprehensive and coordinated blood safety system monitoring activities [4, 21]. Further, training opportunities in this cross-cutting speciality are not offered in any of the benchmarked countries, and as a result this negatively affects the development of haemovigilance systems in those countries [20, 21]. A possible reason for the lack of training opportunities in haemovigilance is that there has not been a focused approach to build capacity by WHO and other development partners unlike what is available for pharmacovigilance.

To improve the implementation and performance of haemovigilance in African countries, effective systems are required to facilitate monitoring and evaluation of donor safety, blood product quality and safety, and transfusion safety. It is necessary for all countries to develop specific legal requirements and regulations to support the establishment and implementation of standardised national haemovigilance safety data collection and reporting with a clear framework of responsibilities and requirements for key institutions. To ensure there is an equivalent level of safety for blood donors, and blood and blood components, it is essential that common standards, definitions and terminologies for blood safety be developed and harmonised between different NRAs, and blood transfusion services with other internationally accepted or recognised standards and requirements [22]. NRAs and blood transfusion services should identify and implement sustainable programs for continuous staff training and capacity building.

Although the GBT+ Blood aims to be comprehensive in assessing the performance of haemovigilance systems, the sub-indicators used only take into account the structures and functions of haemovigilance within the regulatory system context. Other haemovigilance performance indicators such as the number of haemovigilance case reports received by blood centres receiving and analysing haemovigilance data are not taken into account or scored. While the information gathered during the benchmarking was correct at the time of data collection, there are possibilities that NRAs have since updated their systems. Further we, unfortunately, cannot state whether the benchmarking led to improvements in the implementation and performance of haemovigilance systems without an additional benchmarking study. This should be the subject of future work to evaluate whether benchmarking with the WHO GBT+ Blood supports the improvement of haemovigilance system implementation and performance. The authors did not determine the reliability and validity of the scoring system used in the GBT+ Blood. As far as we know, the validity of the scoring method used in the GBT+ Blood has not been tested. We suggest that future studies should check the reliability and validity of the scoring method that is applied by the GBT+ Blood. Finally, future studies should include more NRAs to be able to evaluate the actual discriminative capabilities of the sub-indicator set [23].

## Conclusions

This study provides evidence that the implementation and performance of haemovigilance systems in selected Sub-Saharan African countries is sub-optimal, which may impact donor safety, blood product quality and safety, and transfusion safety. The implementation of legislative provisions for the establishment of haemovigilance is at a nascent stage in most countries. It is clear that training, qualification and capacity building remain fundamental in all aspects of blood safety. This study has further revealed the need to set up targeted and customised technical support coupled with prioritised interventions to strengthen the capacities of NRAs.

## Supplementary information

**Additional file 1**

## Data Availability

The datasets generated and/or analysed during the current study are not publicly available due to the confidential nature of the data, but are available from the corresponding author on reasonable request.

## Abbreviations

AUDA – AMRH: African Union Development Agency – African Medicine Harmonisation Programme
BRN: WHO Blood Regulators Network
FRD: Family Replacement Donation
GBT: Global Benchmarking Tool
HIV: Human Immunodeficiency Virus
ISO: International Standard Organisation
IVD: In-vitro diagnostic
NBTS: National Blood Transfusion Service
NRA: National Regulatory Authority
PIDM: WHO Programme for International Drug Monitoring
VNRD: Voluntary Non-Remunerated Donation
WHA: World Health Assembly
WHO: World Health Organisation
