# Supplementary material for: Implementation and performance of haemovigilance systems in 10 sub-saharan African countries is sub-optimal
Source: BMC Health Serv Res. 2021 Nov 20;21:1258. doi: 10.1186/s12913-021-07235-0 (PMC8605544; doi:10.1186/s12913-021-07235-0)

Implementation and Performance of Haemovigilance Systems in 10 Sub-Saharan African countries is sub-optimal.

**Washington T. Samukange^1, 2^, Verena Klümpers^2^, Manvi Porwal^2^, Linda Mudyiwenyama^3^, Khamusi Mutoti^4^, Noel Aineplan^5^, Helga Gardarsdottir^1^, Aukje K. Mantel-Teeuwisse^1^ and C. Micha Nuebling^2^**

Supplementary Table ST1: Benchmarking steps developed by van Lent and application in this study

| **13 steps by van Lent** | **Application of the steps in this study** |
| --- | --- |
| **1 Determine what to benchmark** | Haemovigilance Function (Blood Regulatory Systems) |
| **2 Form a benchmarking team** | Scientific Officers from the Paul Ehrlich Institut with background in Blood Regulation |
| **3 Choose benchmarking partners** | National Regulatory Authorities in Ethiopia, Kenya, Liberia, Malawi, Nigeria, Uganda, Rwanda, South Africa, Tanzania, and Zimbabwe. |
| **4 Define and verify the main characteristics of the partners** | A mapping exercise of the national blood supply systems and relevant laws in which the NRAs are located was performed. |
| **5 Identify stakeholders** | WHO and group of international experts from members of the WHO Blood Regulators Network |
| **6 Construct a framework to structure the indicators** | The framework was based on that identified in the WHO Global Benchmarking Tool (medicines and vaccines), the WHO Criteria for Assessment of Blood Regulatory Systems and ISO 9004:2018 Quality management — Quality of an organization — Guidance to achieve sustained success |
| **7 Develop relevant and comparable indicators** | Indicators and sub-indicators were drawn from the WHO GBT (medicines and vaccines) and the WHO BRN Assessment Criteria for Blood Regulatory Systems |
| **8 Stakeholders select indicators** | Stakeholders met at WHO, in Geneva in August 2017 and selected indicators from BRN Assessment Criteria to integrate into GBT |
| **9 Measure the set of performance indicators** | Data collection phase was seven months, from February 2018 to August 2018. The same team performed a visit to each NRA to grasp the context and clarify any questions. |
| **10 Analyse performance indicators** | The performance of the haemovigilance systems in 10 countries was compared against the sub-indicators. Reports of this comparison were checked by the other members of the country visit team. |
| **11 Take action: results are presented in a report and recommendations are given** | For each participating NRA, a report was made containing the outcomes of the benchmark for all NRAs. Data was anonymized. Improvement recommendations for each participating NRA were sent in a separate report. |
| **12 Develop relevant plans** | Institutional Development Plans (IDPs) were developed jointly and agreed with each participating NRA. |
| **13 Implement the improvement plans** | Outside the scope of this study. |


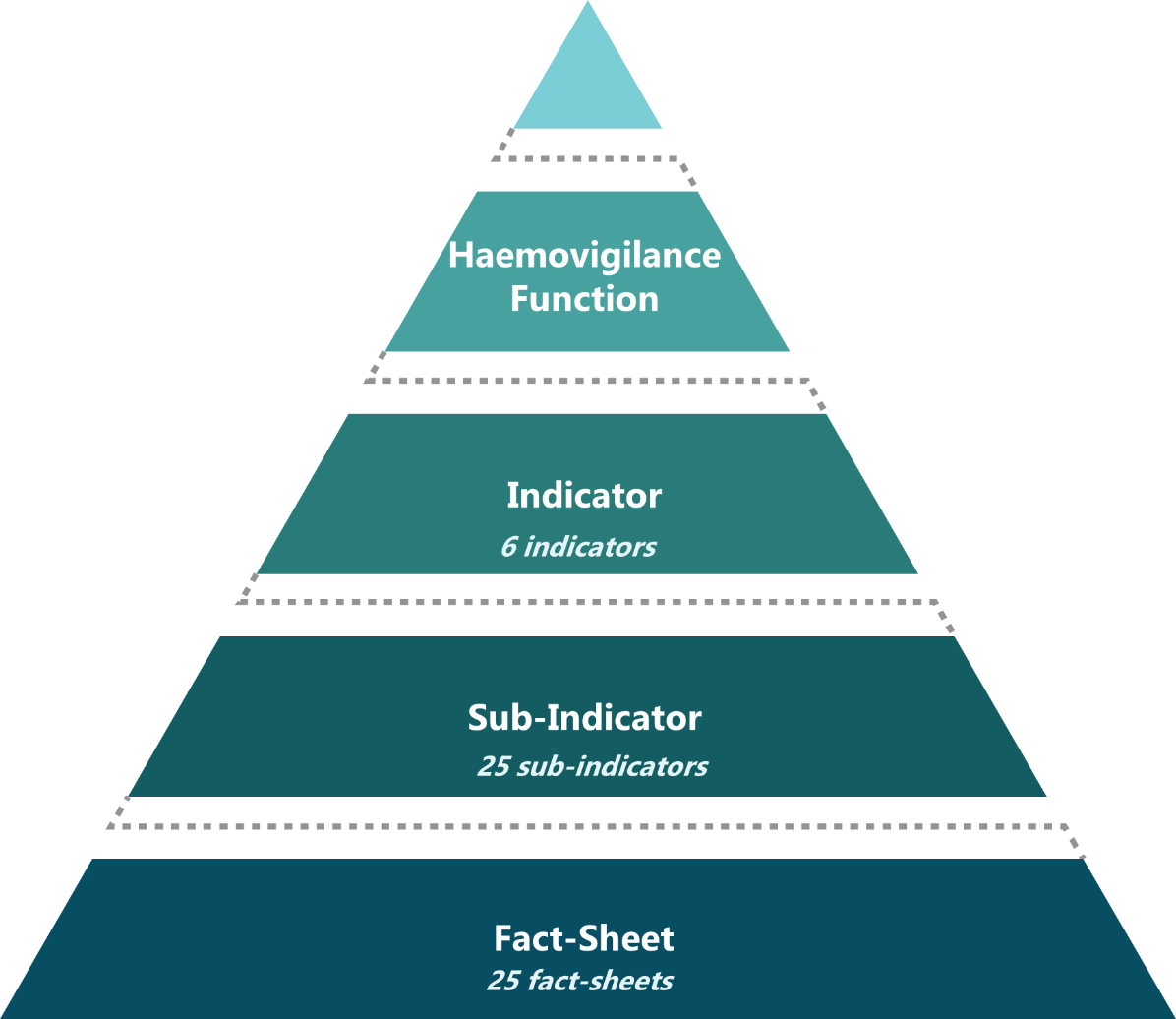


Supplementary Figure SF1: Structure of the Haemovigilance Function in the GBT+ Blood (adapted from the WHO Regulatory Systems Strengthening)

Supplementary Table ST2: Haemovigilance function indicators and sub-indicators in the WHO GBT+ Blood

| **Indicator** | **Sub-indicator** | **Maturity Level** |
| --- | --- | --- |
| VL01 Legal provisions, regulations and guidelines required to define regulatory framework of vigilance. | VL01 Legal provisions, regulations and guidelines required to define regulatory framework of vigilance. | **1** |
| VL01 Legal provisions, regulations and guidelines required to define regulatory framework of vigilance. | VL01.02: Legal provisions, and/or regulations require the manufacturers and/or MAH to set up a vigilance system of their medical products and periodically report vigilance data, including zero events, to the NRA. | **1** |
| VL01 Legal provisions, regulations and guidelines required to define regulatory framework of vigilance. | VL01.03: Regulations encourage distributors, importers, exporters, health- care institutions, other stakeholders and consumers to report the vigilance events to the MAH and/or NRA. | **1** |
| VL01 Legal provisions, regulations and guidelines required to define regulatory framework of vigilance. | VL01.04: The legal provisions and/or regulations allow NRA to require manufacturers and/or MAH to conduct specific studies on safety and efficacy under specific conditions. | **2** |
| VL01 Legal provisions, regulations and guidelines required to define regulatory framework of vigilance. | VL01.05: Legal provisions, regulations and/or guidelines require manufacturers and/or marketing authorization holders to designate a Qualified Person responsible for vigilance. | **3** |
| VL01 Legal provisions, regulations and guidelines required to define regulatory framework of vigilance. | VL01.06: There are guidelines for planning, conducting (including monitoring) and reporting of vigilance activities. | **3** |
| VL01 Legal provisions, regulations and guidelines required to define regulatory framework of vigilance. | VL01.07: Legal provisions and/or regulations allow recognition and/or reliance on vigilance-relevant decisions, reports or information from other countries, regional and/or international bodies. | **1** |
| VL02 Arrangement for effective organization and good governance. | VL02.01: There is a defined structure with clear responsibilities to conduct vigilance activities. | **2** |
| VL02 Arrangement for effective organization and good governance. | VL02.02: Collaboration between all stakeholders relevant to medical products vigilance is in place. | **3** |
| VL03 Human resources to perform vigilance activities. | VL03.01: Enough competent staff (education, training, skills and experience) are assigned to perform vigilance activities. | **3** |
| VL03 Human resources to perform vigilance activities. | VL03.02: Respective job descriptions are established and regularly updated with inclusion of duties, functions, responsibilities and necessary competencies. | **3** |
| VL03 Human resources to perform vigilance activities. | VL03.03: Training plan developed, implemented and updated at least once a year. | **3** |
| VL03 Human resources to perform vigilance activities. | VL03.04: The NRA performs and maintains records of staff training activities and training effectiveness verification. | **3** |
| VL04 Procedures established and implemented to perform vigilance activities. | VL04.01: Risk approach is considered throughout different vigilance activities, including timely response to safety signals. | **3** |
| VL04 Procedures established and implemented to perform vigilance activities. | VL04.02: Vigilance procedures and tools are in place for collection, assessment, investigation and interpretation of safety issues. | **3** |
| VL04 Procedures established and implemented to perform vigilance activities. | VL04.03: Staff access to information resources relevant to vigilance processes (e.g. safety information sources and reference materials) is ensured. | **3** |
| VL04 Procedures established and implemented to perform vigilance activities. | VL04.04: Expert committee(s) exists to review serious vigilance events. | **3** |
| VL04 Procedures established and implemented to perform vigilance activities. | VL04.05: Assessment of the risk/benefit balance of medical products is regularly conducted considering vigilance data. | **3** |
| VL04 Procedures established and implemented to perform vigilance activities. | VL04.06: The development and implementation of a proactive monitoring programme to promote adherence to vigilance by the NRA. | **4** |
| VL04 Procedures established and implemented to perform vigilance activities. | VL04.07: Standard procedures exist and are implemented for enforcement of the national vigilance programme. | **4** |
| VL05 Mechanism exists to promote transparency, accountability and communication | VL05.01: Mechanism for regular feedback to all stakeholders on vigilance events exists and is complemented with a risk communication plan. | **4** |
| VL05 Mechanism exists to promote transparency, accountability and communication | VL05.02: Vigilance activities and feedbacks are appropriately communicated to the public community. | **4** |
| VL05 Mechanism exists to promote transparency, accountability and communication | VL05.03: Vigilance data and findings are shared with relevant regional and international partners. | **3** |
| VL06 Mechanism in place to monitor regulatory performance and output. | VL06.01: Vigilance information is used to amend or issue regulatory decisions and consequent actions in timely manner. | **3** |
| VL06 Mechanism in place to monitor regulatory performance and output. | VL06.02: Performance indicators for vigilance activities are established. | **4** |

Supplementary Figure SF2: WHO GBT Performance Maturity Levels, adapted with permission from WHO Regulatory Systems Strengthening [6]


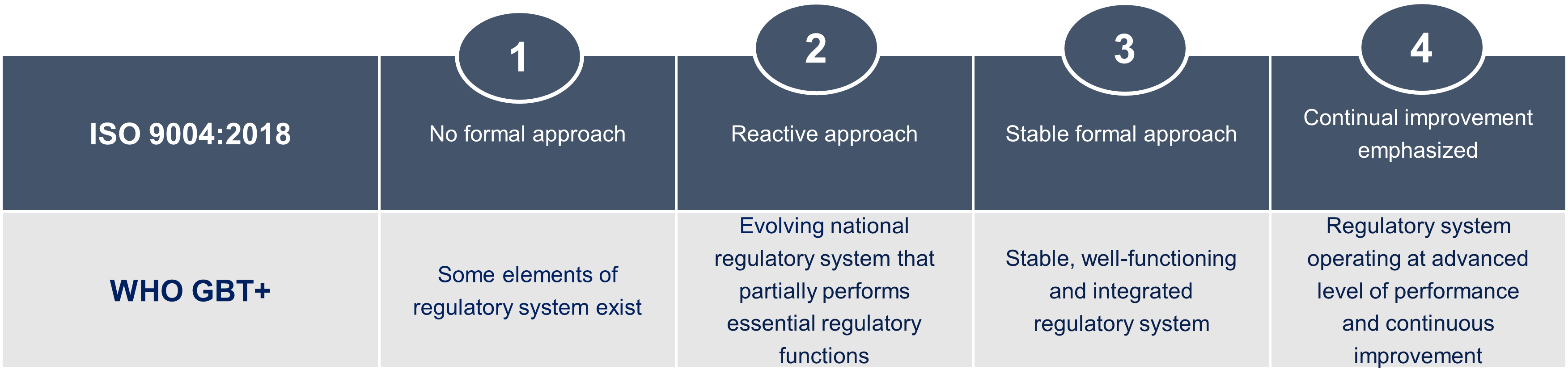

Supplement: Supplementary file 1 — Additional file 1 [file 12913_2021_7235_MOESM1_ESM.docx]
